# Subunit Positioning and Stator Filament Stiffness in Regulation and Power Transmission in the V_1_ Motor of the *Manduca sexta* V-ATPase^☆^

**DOI:** 10.1016/j.jmb.2013.09.018

**Published:** 2014-01-23

**Authors:** Stephen P. Muench, Sjors H.W. Scheres, Markus Huss, Clair Phillips, Olga Vitavska, Helmut Wieczorek, John Trinick, Michael A. Harrison

**Affiliations:** 1 School of Biomedical Sciences, Faculty of Biological Sciences, University of Leeds, Leeds LS2 9JT, UK; 2 MRC Laboratory of Molecular Biology, Hills Road, Cambridge CB2 0QH, UK; 3 Abteilung Tierphysiologie, Fachbereich Biologie/Chemie, Universität Osnabrück, 49069 Osnabrück, Germany; 4 School of Molecular and Cellular Biology, Faculty of Biological Sciences, University of Leeds, Leeds LS2 9JT, UK

**Keywords:** 3D, three dimensional, EM, electron microscopy, vacuolar membrane, H^+^-ATPase, cryo-electron microscopy

## Abstract

The vacuolar H^+^-ATPase (V-ATPase) is an ATP-driven proton pump essential to the function of eukaryotic cells. Its cytoplasmic V_1_ domain is an ATPase, normally coupled to membrane-bound proton pump V_o_ via a rotary mechanism. How these asymmetric motors are coupled remains poorly understood. Low energy status can trigger release of V_1_ from the membrane and curtail ATP hydrolysis. To investigate the molecular basis for these processes, we have carried out cryo-electron microscopy three-dimensional reconstruction of deactivated V_1_ from *Manduca sexta*. In the resulting model, three peripheral stalks that are parts of the mechanical stator of the V-ATPase are clearly resolved as unsupported filaments in the same conformations as in the holoenzyme. They are likely therefore to have inherent stiffness consistent with a role as flexible rods in buffering elastic power transmission between the domains of the V-ATPase. Inactivated V_1_ adopted a homogeneous resting state with one open active site adjacent to the stator filament normally linked to the H subunit. Although present at 1:1 stoichiometry with V_1_, both recombinant subunit C reconstituted with V_1_ and its endogenous subunit H were poorly resolved in three-dimensional reconstructions, suggesting structural heterogeneity in the region at the base of V_1_ that could indicate positional variability. If the position of H can vary, existing mechanistic models of deactivation in which it binds to and locks the axle of the V-ATPase rotary motor would need to be re-evaluated.

## Highlights

• Dissociation of vacuolar H^+^-ATPase domains deactivates its V_1_ motor.
• V_1_ has one “open” catalytic site linked to the stator filament bound by subunit H.
• Movement of subunit H to prevent rotary catalysis is possible.
• Three stator filaments project from deactivated V_1_, indicating inherent stiffness.
• This work gives new insight into energetic coupling and control in V-ATPases.

## Graphical abstract

**Figure:**
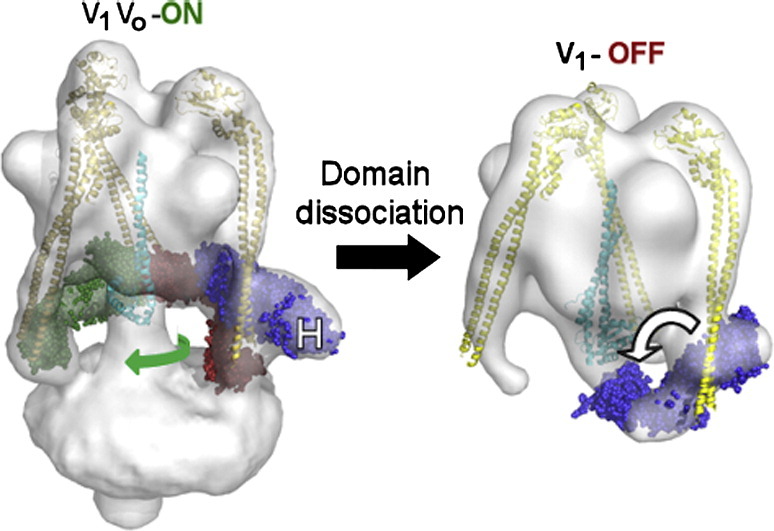

**Legend:**
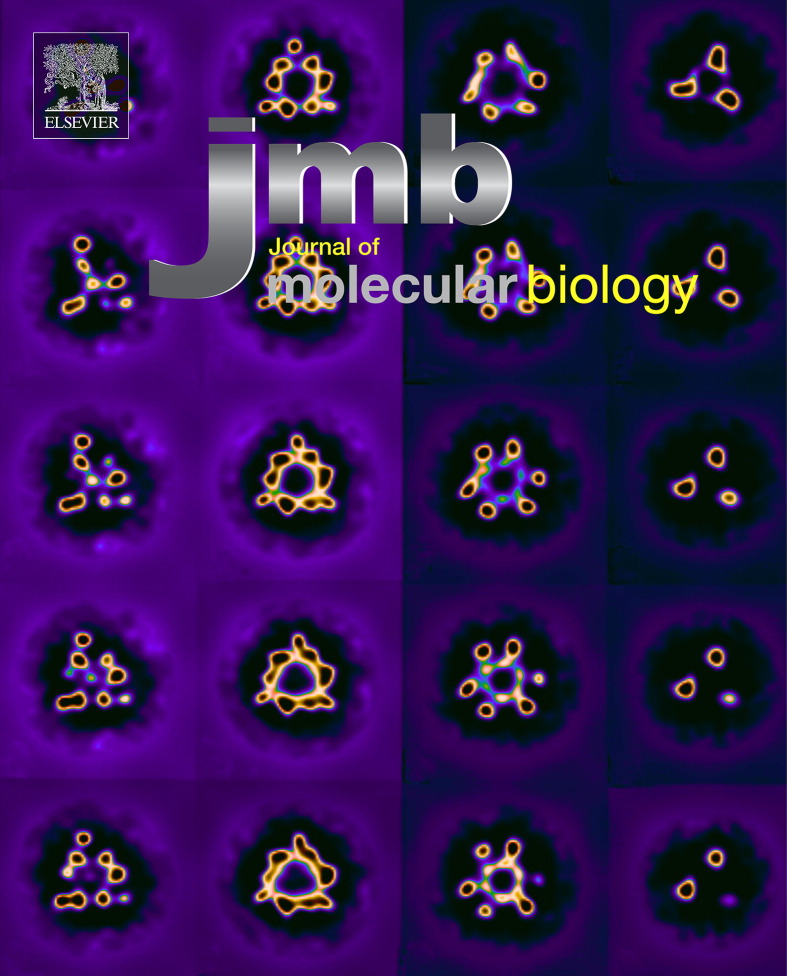
In some organisms, the V_1_ domain of the vacuolar H^+^-ATPase rotary proton pump can be reversibly decoupled from the membrane, temporarily switching off proton translocation when ATP conservation is the priority. The cover image shows sections through an ~ 20-Å-resolution electron density map of deactivated V_1_, generated by cryo-electron microscopy, that reveals the conformational changes that accompany catalytic silencing.

## Introduction

Vacuolar H^+^-ATPases (V-ATPases) are complex membrane-bound rotary molecular motors that pump protons against membrane potential, fuelled by the free energy of ATP hydrolysis. They play central roles in virtually all eukaryotic cells, such as acidification of intracellular compartments and energisation of secondary active transport [1], [2]. V-ATPases share a common bi-domain architecture and rotational mechanism with the F_1_F_o_-ATPase (ATP synthase) and archaeal A_1_A_o_-ATPase [3] but are substantially larger and more complex, with several unique subunits. The extra subunits are proposed to mediate regulation in response to factors such as changes in glucose status [4], [5] or hormonal signals [6].

**Fig. 1:**
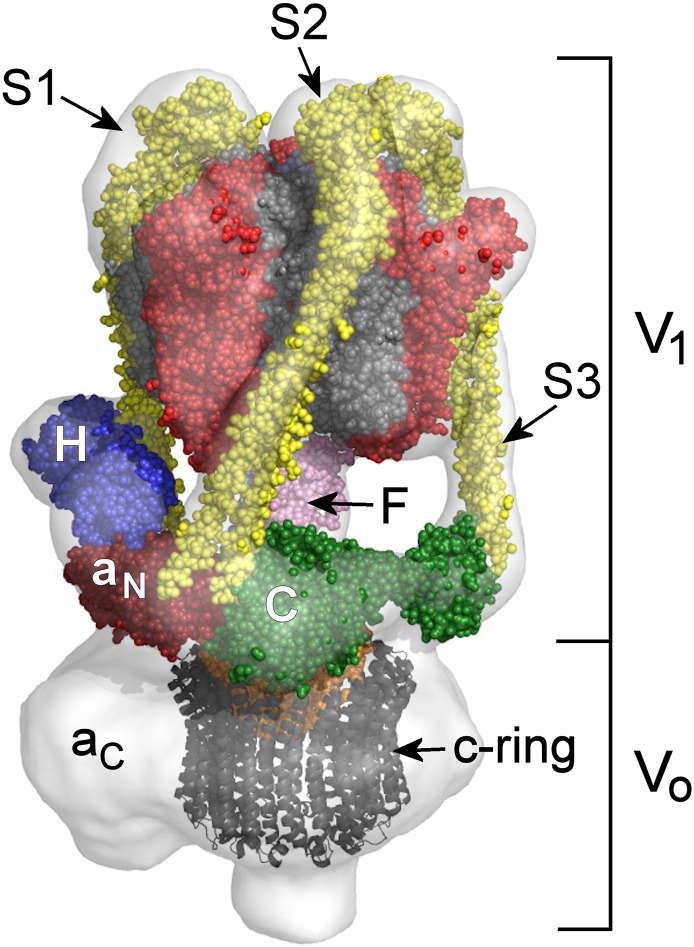
Model of the V-ATPase. A mosaic model of the V-ATPase was produced by fitting crystal structures of homologous subunits to the 17-Å-resolution reconstruction of the *M*. *sexta* V-ATPase [7] in-line with biochemical and biophysical data. Subunits A, B and F (PDB ID 3W3A[8]) are red, grey and pink. Subunit D that forms the axle of the V_1_ motor is not visible. The subunit E/G stator filaments (yellow; PDB ID 3K5B[9]) are numbered S1–S3 as in Ref. [3]. The inter-domain collar contains *a*_N_ (red; PDB ID 3RRK[10]), which is the N-terminal domain of subunit *a*, and subunits H (blue; PDB ID 1HO8[11]) and C (green; PDB ID 1U7L[12]). In the membrane domain V_o_, the subunit c ring {represented here by its decameric NtpK homologue (grey ribbon format; PDB ID 2BL2[13])} is plugged by subunit *d* (orange; PDB ID 1R5Z[14]). There is currently no high-resolution structure for the membrane domain of subunit *a* (*a*_C_).

In the eukaryotic V-ATPase (Fig. 1), subunits A–H (with stoichiometry A_3_B_3_CDE_3_FG_3_H [15]) form the ~ 660-kDa V_1_ domain, with the ATPase motor consisting of an (AB)_3_ hexamer coupled to a central subunit D/subunit F rotor axle [16]. This axle is coupled to a decameric ring of *c* subunits [13] via subunit *d* in the V_o_ membrane domain (Fig. 1). Proton translocation results from rotation of the D/F/*d*/*c*_10_ complex relative to V_o_ subunit *a* by a mechanism that remains unclear. For productive movement to occur, subunit *a* must be fixed to the (AB)_3_ motor as part of a mechanical stator. In F_1_F_o_-ATPase, this stator contains a single, apparently rigid filament linking the ATPase motor to subunit *a* [17]. In contrast, the A_1_A_o_-ATPase stator has two separate filaments linked by *a*_N_, the cytoplasmic N-terminal domain of its subunit *a* homologue [18], [19]. The V-ATPase stator has a more complex arrangement of three subunit E/G filaments that are all linked to the membrane domain of subunit *a* via a collar feature that partly encircles the region between V_1_ and V_o_ (Fig. 1) [7], [20], [21]. In addition to *a*_N_, this collar contains subunits C and H, polypeptides unique to the V-type complex and implicated in its regulation. Subunit C and the *a*_N_ domain show remarkably similar folds but not sequences [10], [12].

In some cells, V_1_ can rapidly but reversibly dissociate from V_o_ when energy levels are low, for example, during insect larval moulting [6] or as a result of glucose depletion [4], [22]. This is accompanied by silencing of ATP turnover by detached V_1_ [4], [6]. The mechanism of dissociation remains uncertain but appears to involve phosphorylation of subunit C [23], [24], [25]. According to this model, signalling through subunit C destabilises the stator, allowing separation of V_1_ from V_o_. In yeast, ATPase silencing in detached V_1_ requires subunit H [26], [27]. H can interact with subunit F only in the detached domain [28], consistent with its repositioning after domain dissociation. In larval *Manduca*, each V_1_ turns over, on average, one ATP before becoming catalytically inactive [29], suggesting that a rotational step is required for transition to the catalytically inactive configuration. In an alternative model, futile ATP turnover is prevented by subunit F extending and tethering the rotor axle to the catalytic AB interface [16] to prevent rotational stepping, analogous to a proposed role for the ε-subunit in F_1_F_o_-ATPase [30]. Crucially, a lack of structural information about the physiologically inactivated V_1_ in its native state severely limits our understanding of the mechanism of dissociation and deactivation. This also extends to our understanding of the stages in the re-engagement of the collar subunit C that must precede domain re-coupling.

There has been debate recently about the intrinsic flexibility of the subunit E/G filaments within the V-ATPase stator. Because their ATPase and H^+^ pump functions are symmetrically and therefore stoichiometrically mismatched (3 ATP *versus* 10 H^+^), direct coupling is unlikely. Instead, an elastic power transmission model [31] where flexibility buffers energy transfer between V_1_ and V_o_ has been postulated. The question remains whether the EG filaments perform this function. Absence of peripheral stalks in three-dimensional (3D) reconstructions of yeast or *Manduca* V_1_ from negative-stain electron microscopy (EM) data [32], [33] implies intrinsic flexibility that allows the stalks to collapse once disconnected. More recently, comparison of crystallographic EG structures by normal mode analysis also suggests flexibility, but not in the direction that would necessarily be imposed by the rotational mechanism [34].

Here, we report the first complete 3D reconstruction of eukaryotic V_1_, deactivated and in complex with subunit C. These models, determined by cryo-EM, show new detail that allows direct comparison between deactivated and active V-ATPases. This reveals that the EG stator filaments undergo essentially no structural rearrangement as a consequence of V_1_ dissociation, indicating that they have inherent stiffness consistent with a role in elastic power transmission. Subunit H is poorly resolved, which could indicate conformational heterogeneity in the region of the complex that forms a major part of the stator linkage in the intact enzyme. These observations have implications for models of energetic coupling and regulation of the V-ATPase.

## Results

### Preparation of V_1_ and reconstitution with recombinant subunit C

**Fig. 2:**
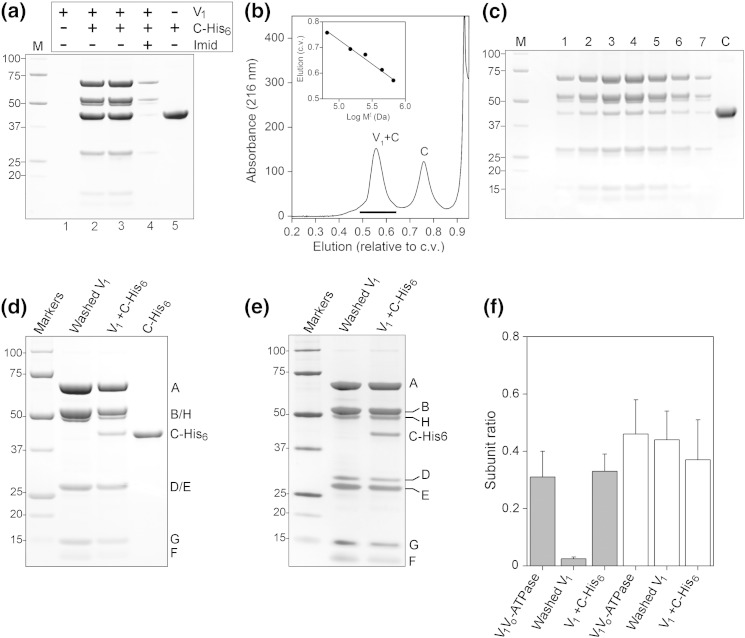
The *M*. *sexta* V_1_ domain: Purification and reconstitution with subunit C. (a) SDS-PAGE of V_1_ re-association with recombinant C-His_6_. V_1_ was able to bind Ni^2 +^-NTA-conjugated resin only after incubation with C-His_6_ (lane 2) or to resin pre-bound with C-His_6_ (lane 3). V_1_ alone showed no non-specific binding to the resin (lane 1), and binding was inhibited by the presence of competing 300 mM imidazole, indicating His-tag dependence of binding. Lane 5 shows a His_6_-C binding control. (b) Separation of V_1_^+ C^ from unbound C-His_6_ by size-exclusion chromatography. The V_1_/C-His_6_ mixture was separated on a Superose-6 column. Monitory absorbance at 216 nm showed two peaks corresponding to V_1_^+ C^ and free C-His_6_. Column calibration (inset) as outlined in Materials and Methods gave approximate masses of 660 kDa and 50 kDa for the mid-points of the two elution peaks. The horizontal line indicates the range of fractions analysed in (c). (c) SDS-PAGE analysis of fractions eluting from the chromatographic separation shown in (b). The apparent masses of polypeptides in the eluted protein are 69 kDa (subunit A), 56 kDa (subunit B), 55 kDa (subunit H), 26 kDa (subunit E), 12 kDa (subunit F) and 15 kDa (subunit G). C-His_6_ was evident in all fractions at approximately equal stoichiometry with the H subunit. V_1_^+ C^. Subunit D (~ 28 kDa) stained very weakly with Coomassie. (d) SDS-PAGE analysis of purified V_1_ washed with 25% methanol to remove residual subunit C, purified V_1_ reconstituted with C-His_6_ and purified recombinant C-His_6_. (e) SDS-PAGE analysis of V_1_ and V_1_^+ C^ stained with silver to show the presence of subunit D. (f) densitometry of C and H subunits after SDS-PAGE separation. Levels of subunit C (shaded histogram) and H (white) are expressed relative to the intensity of subunit A staining for the intact V-ATPase, washed V_1_ and V_1_ reconstituted with C-His_6_.

When purified as described in Ref. [35] after *in vivo* inactivation, tobacco hornworm (*Manduca sexta*) V_1_ contains seven polypeptide types: A, B, E, G and H that form part of the stator in the intact enzyme, with rotor subunits D and F. The V_1_ detachment/inactivation process results in almost complete loss of subunit C [36], but the inactive complex retains the ability to bind this polypeptide. Any residual C is removed by incubation with methanol (25%) prior to the final step in the purification process. Since C is generally required for active complex [37], re-binding may be presumed to be an early step in the reactivation process *in vivo*. Incubation of V_1_ with ~ 5-fold molar excess of recombinant His_6_-tagged subunit C resulted in retention of V_1_ by chelated metal affinity resin (Fig. 2a), indicating strong re-binding of C. V_1_ did not bind in the absence of C-His_6_ (Fig. 2a, lane 1), and binding of the re-associated complex was strongly inhibited by inclusion of high concentration of competing imidazole, indicating His-tag dependence. V_1_ recovery was essentially the same regardless of whether C was pre-bound to the affinity resin (Fig. 2a, lane 2) or incubated with V_1_ prior to pull-down (lane 3). To examine the efficiency of binding, we separated V_1_ incubated with C by size-exclusion chromatography (Fig. 2b and c). V_1_ eluted from the column as a single discrete peak, with each fraction containing C-His_6_. Densitometry of the resulting SDS-PAGE gels indicated an apparent stoichiometry of approximately 1:3 with subunit A and of 1:1 with subunit H (Fig. 2f), not significantly different from the relative levels in the intact holoenzyme. Calibration of the column (Fig. 2b, inset) gave an approximate mass of ~ 660 kDa for V_1_^+ C^, consistent with an (AB)_3_(EG)_3_CDFH composition. Unbound C-His_6_ eluted with an apparent mass of ~ 50 kDa.

Comparison of V_1_ and re-purified V_1_^+ C^ after separation on SDS-PAGE shows the characteristic presence of A (69 kDa), B (56 kDa), E (26 kDa), F (14 kDa), G (13.5 kDa) and H (55 kDa) subunits, with C-His_6_ evident in V_1_^+ C^. Subunit D (~ 28 kDa) appears to stain very weakly with Coomassie (Fig. 2d), but its presence can be detected by staining with silver (Fig. 2e). Densitometry of SDS-PAGE separations of V_1_, V_1_^+ C^ and the V-ATPase holoenzyme indicate that levels of subunit H are not significantly different in the three complexes (approximately 1:3 with subunit A; Fig. 2f).

### EM of *M*. *sexta* V_1_

**Fig. 3:**
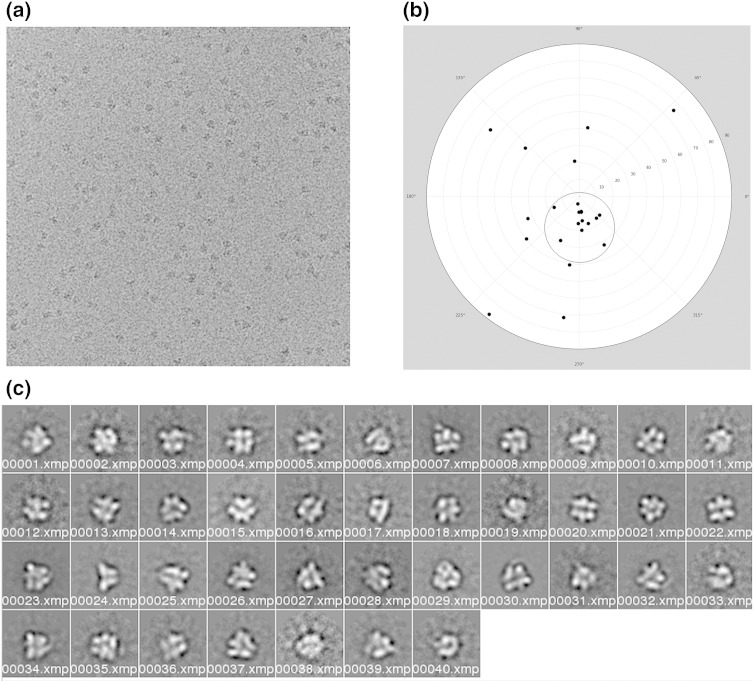
Cryo-EM of V_1_^+ C^. (a) Example of cryo-EM grid. (b) Tilt-pair plot for the V_1_ reconstruction generated using the tilt validation protocol in EMAN2 [38] and using *C*1 symmetry (e2tiltvalidate.py). The circle is centred on the expected tilt angle of 15°, with an RMSD tilt angle of 9°. The relative proportion of tilt pairs that are within the circle is 0.68. (c) Individual classes of V_1_^+ C^ generated using XMIPP.

At 1 mg/ml protein, a high density of single V_1_ particles was visible on negative-stain grids, indicating monodispersity at this concentration (an example is shown in Supplemental Fig. S1a). Both V_1_ and V_1_^+ C^ distributed evenly in thin ice on holey carbon grids (Fig. 3a), allowing a total of ~ 42,000 randomly oriented particles to be picked. After removing particles that were of poor quality and were not stable during maximum-likelihood refinement, the resulting stacks contained 16,500 V_1_ and 10,746 V_1_^+ C^ particles. After classification, image averages showed a range of views of the V_1_^+ C^ complex (Fig. 3c), consistent with random orientation of the particles in ice.

A refined negative-stain model in which stator filaments are not resolved (Supplemental Fig. S1d) was low-pass filtered to 100 Å and used to generate four randomly different seed structures for subsequent multi-reference refinement using MLF3D [39] in the XMIPP package [40]. After simultaneous refinement of the four seeds, one of the four refined models contained more than half the particles and showed distinct features; the other three models were assumed to contain degraded particles or noise. In order to verify the model from the most populated class, we ran MLF3D twice more with different random seeds. On each occasion, despite the starting model being relatively featureless, the same reconstruction was returned. Analysis of the particle distribution showed that 85% of the particles were stable and consistently grouped into the same model. The resolution of this reconstruction was estimated at 30 Å for the SSNR cutoff that is intrinsic to the MLF3D procedure. The resulting model was filtered to 50 Å resolution and used as an initial model for Bayesian refinement in the RELION programme [41] of both the native V_1_ and the V_1_^+ C^ data. The estimated resolutions for the refined maps, which were based on gold-standard Fourier shell correlations [42], were 22 Å for V_1_ and 20 Å for V_1_^+ C^. Tilt-pair analysis confirmed the absolute hand of the reconstruction and the validity of information in it (Fig. 3b) [43].

### Structure of V_1_ reconstituted with subunit C

**Fig. 4:**
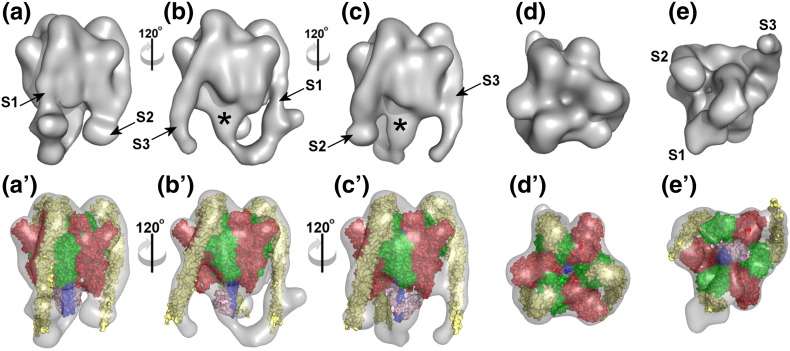
3D reconstruction of the V_1_^+ C^ domain. (a–c) Side views of V_1_^+ C^ rotated in 120° steps. The end of the complex facing the membrane is at the bottom. The subunit E/G stator filaments are numbered S1–S3. (d and e) End-on views of the cytoplasmic surface and V_o_-binding ends, respectively. The rotor axle is indicated by the asterisk. (a′–e′) Corresponding views of V_1_^+ C^ with crystal structures of homologous subunits fitted using Chimera. Subunits A, B, D and F (PDB ID 3W3A) are red, green, purple and pink. The EG heterodimer (PDB ID 3K5B) is yellow. The electron density map is contoured to 1.5σ.

The 3D model of V_1_^+ C^ shows an essentially globular structure that is ~ 185 Å long (perpendicular to the plane of the membrane) and ~ 145 Å wide at its maximum (Fig. 4 and Supplemental Movie 1). The core, containing the catalytic A and B and axle D/F subunits, shows the approximate 3-fold symmetry typical of this region of rotary ATPases [3]. The crystal structures of (AB)_3_DF [8], [16] and (AB)_3_ [44] complexes from the homologous *Thermus thermophilus* A_1_ domain can be fitted into the *Manduca* V_1_^+ C^ reconstruction such that they do not, at any point, protrude significantly from the density map (Fig. 4a′–e′; Supplemental Movie 1). The (AB)_3_ complex (PDB ID 3GQB) [44] is a lower scoring fit to the reconstruction than (AB)_3_DF crystal structures (PDB IDs 3W3A and 3A5D3W3A3A5D) [8], [16]. 3GQB lacks the subunit D/F rotor axle and has three equivalent, symmetrical AB units. Both 3W3A and 3A5D3W3A3A5D show some asymmetry in their AB units imposed by the DF axle and have one unoccupied catalytic site that is in a more “open” conformation compared to the other two “closed” ADP-bound sites. The “non-homologous region” of each A subunit fits into the three knob-like densities projecting at 120° intervals from complex. A well-defined central stalk projecting ~ 50 Å downwards (asterisk in Fig. 4) is the axle of V_1_. It accommodates crystal structures of the subunit D helical coiled coil [16], [45] with the 14-kDa F subunit at its base.

**Fig. 5:**
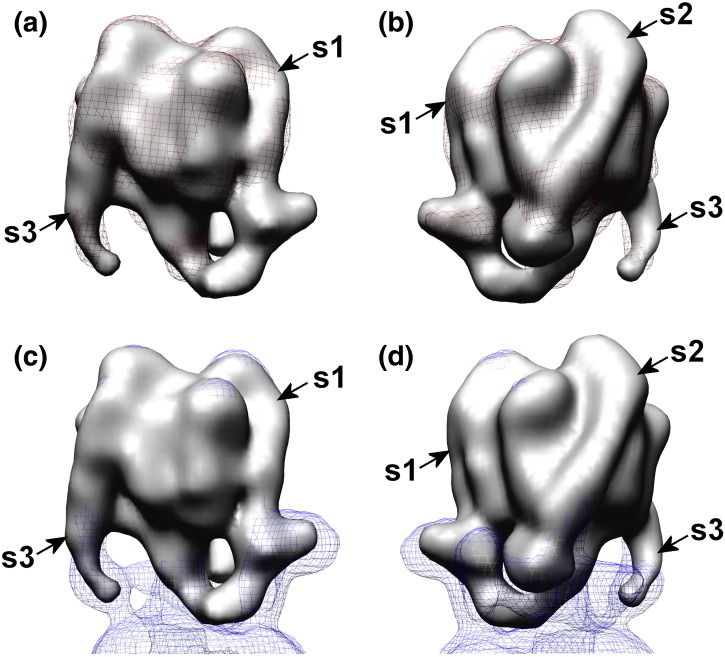
Comparison of V_1_^+ C^ and V_1_ reconstructions. Overlays of surface-rendered V_1_^+ C^ with (a and b) V_1_ (red mesh) and (c and d) V-ATPase [7] (blue mesh) reconstructions contoured to the same level (1.5σ). (b) and (d) are rotated 180° with respect to (a) and (c). See also Supplemental Fig. S2.

All three of the stator filaments observed in the intact V-ATPase are also visible in V_1_^+ C^ (Fig. 4), hooking over the top of the complex and passing down the surface of (AB)_3_ to project as unsupported ~ 50-Å filaments. In intact V-ATPase, all three filaments connect to a collar structure that almost completely surrounds the middle of the complex [7], [20], [21]. The filament designated S1 directly connects to subunit *a* and subunit H in the collar, whereas S2 (also found in the A-ATPases [3], [18], [46]) is linked to S1 via *a*_N_. S3, exclusively found in the V-ATPase, is connected to S2 via subunit C [7], [20], [21]. Overlay of the reconstruction of V_1_ without C with that of V_1_^+ C^ shows very similar core structures with projecting stator filaments that are largely superimposable (Fig. 5a and b). The most significant difference between the two reconstructions is that a weak density linking S1 to the central axle in V_1_^+ C^ is not evident in V_1_ without C when contoured at 1.5σ, discussed below. The reconstruction of V_1_^+ C^ can also be superimposed onto the *Manduca* holoenzyme model with almost complete overlap of the core (AB)_3_ region and stator filaments (Fig. 5c and d). For comparison, side-by-side views of the reconstructions are also provided in Supplemental Fig. S2. Each stator filament is a subunit EG heterodimer comprising a right-handed α-helical coiled coil terminating at the globular C-terminal domain of E [19]. Different crystal forms of EG have been observed [9], [34], [47], indicating that the molecule (and therefore each stator filament) has some inherent flexibility. Here, however, the room-temperature configurations of the stator filaments trapped by freezing will be their minimal energy states. The ability here to resolve them extending beyond the base of (AB)_3_ indicates that they must have some inherent stiffness. They therefore most likely act as flexible rods linking the V-ATPase domains, rather than being analogous to cables (as might occur, e.g., in a tensegrity-type model of domain linkage).

In general, all four of the available crystal structures for the EG heterodimer [9], [34], [47] could be accommodated by the S1–S3 stalks in the V_1_^+ C^ reconstruction (Supplemental Fig. S3). Qualitatively, the shape of S1 is similar in V_1_^+ C^ and the holoenzyme and is distinct from that of S2 and S3, with a relatively steep ~ 10° off-vertical angle (Fig. 5c and d; Supplemental Fig. S3). This has been proposed to accommodate the EG configuration in which the segments of E appear straightened (PDB ID 4DL0) [47]. Both S2 and S3 adopt ~ 25° angles in V_1_^+ C^, which for S3 is the same as observed in the holoenzyme (Fig. 5c and d). S2 in V_1_^+ C^, however, has ~ 7° greater angle (displaced along the azimuth, not radially) than observed in the V-ATPase (evident in Fig. 5d and detailed in Supplemental Fig. S3b–d), suggesting that breakdown of the *a*_N_-S2-C three-way contact point allows limited repositioning of S2. Because of its greater curvature, the “PS2” form of EG (PDB ID 3V6I) [34] is a better fit to S2 and S3 than the other forms (Supplemental Fig. S3a). It follows that, since the “PS2” EG form is the best fit to the “relaxed” S2, we can speculate that this is the lowest energy form of the heterodimer. However, it should be noted that higher resolution would be required for complete confidence in assigning the correct EG configuration to S2 and S3. The quality of the fitting is particularly limited at the free ends of each stator filament, perhaps indicating disorder at the tips of the filaments.

**Fig. 6:**
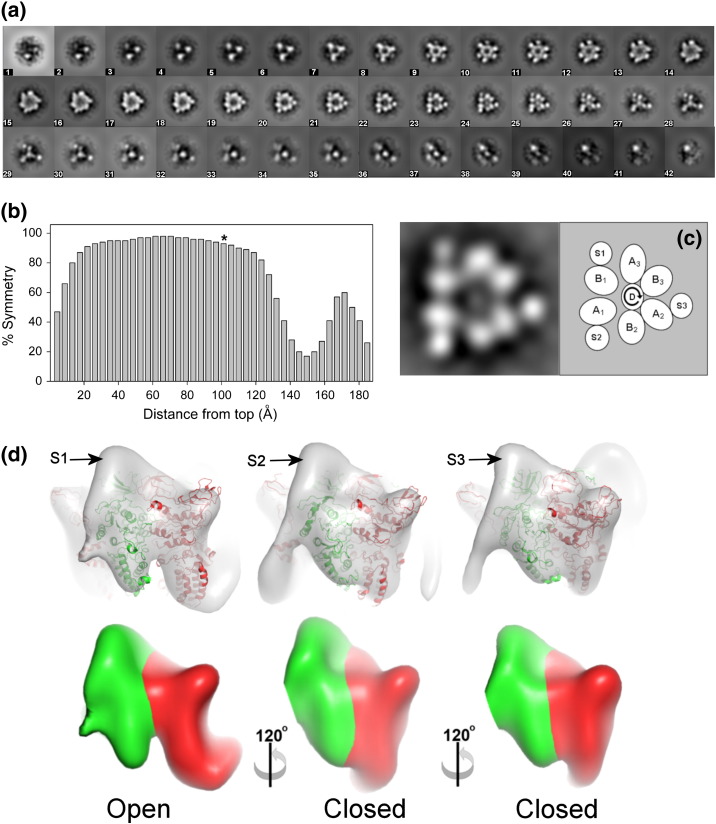
Asymmetry in V_1_^+ C^. (a) Sections through the V_1_^+ C^ map. Section 1 is the top of the complex, equivalent to the cytoplasmic end in the holoenzyme, and section 42 is the membrane-proximal end. Each section, taken perpendicular to the long axis of the complex, is 4.4 Å thick. Stator filaments S1–S3 are labelled as in Fig. 4. (b) Deviation from 3-fold symmetry in the electron density map sections shown in (a). The asterisk indicates section 23, shown expanded in (c) with a schematic for the arrangement of subunits. The curved arrow indicates the direction of axle rotation during ATP hydrolysis. (d) (upper row) Fitting of structures of subunit A/B catalytic units from PDB ID 3W3A[8] into the corresponding densities in the V_1_^+ C^ model, and (lower row) surface representation of regions corresponding to A and B in V_1_^+ C^. In each case, subunit A is red and subunit B is green. “Closed” sites occupied by ADP are best fits to the AB regions bound by stator filaments S2 and S3, whereas the “open” unoccupied site is the best fit to the AB region associated with S1. In the lower row, A/B boundaries are estimated on the basis of the crystal structure fitting.

Departures from the exact 3-fold symmetry are apparent in sections through the V_1_^+ C^ map taken perpendicular to the long axis to the complex (Fig. 6). Greatest asymmetry is observed in sections ~ 140–160 Å from the top of the complex (Fig. 6b), this region having weak density linking S1 to the axle. The significance of this density is discussed below. Substantial asymmetry is also seen at the apex of the complex where the ends of each stator stalk (comprising the C-terminal domain of subunit E) attach to the B subunits (as seen in Fig. 4). Although the “core” part of the complex shows a high degree of symmetry across sections covering ~ 100 Å, closer examination reveals localised asymmetry in some features. In particular, the lower third of the “core” (sections between ~ 90 and ~ 125 Å from the top) shows variable spacing between AB units and each stator stalk and an asymmetric location of the subunit D axle (Fig. 6c). The section highlighted in Fig. 6c, ~ 100 Å from the top of the complex, corresponds to the region at which subunit A segments that are homologous to the conserved DELSEED motif in the F_1_-ATPase β-subunit are closest to the axle [16]. The AB subunits between S1 and S2 (shown schematically as A1/B1 in Fig. 6c) are ~ 20% further apart compared to the other catalytic units and further from the axle. Unequal spacing between the stator filament densities in this section reflects the steeper angle of S1 compared to S2 or S3. The greater spacing between the densities corresponding to subunits A and B is apparent when viewed surface rendered in 3D (Fig. 6d). In the partial A_1_ domain crystal structures, greatest separation at the region homologous to the DELSEED loop occurs between the A and B subunits that share an unoccupied catalytic site [8], [16]. When fitting the asymmetric (AB)_3_DF crystal structure (PDB ID 3W3A) to V_1_^+ C^ using Chimera, the highest score is achieved if this “open” catalytic AB unit is fitted to the A1/B1 region of the reconstruction that has more widely spaced densities (Fig. 6d, upper row). The EM data therefore indicate that, in its resting state, dissociated V_1_ contains AB units in at least two different conformations. Biochemical data indicate that these are likely to result from different nucleotide-bound states [20]. Measurements made with *Manduca* V_1_ show ~ 2 mol ADP per mole protein, compared to a molar ratio of ~ 0.3 for the V-ATPase holoenzyme [29]. Thus, at least one active site in V_1_ is in the empty “open” state of the binding change model of ATPase catalysis [48], and we therefore propose that the AB unit bound to S1 is in this “empty” state. These data show that V_1_ domains inactivated *in vivo* via physiological signals come to rest in a homogeneous state, since such subtle differences in shape could not otherwise be resolved by the methods used here.

The region at the base of the complex that has greatest asymmetry also shows differences between the V_1_ and V_1_^+ C^ models so that different and distinct models were generated (Fig. 5; Supplemental Fig. S2). In particular, a weak density linking S1 to the rotor axle evident in V_1_^+ C^ (Fig. 4) is absent from the V_1_ reconstruction (Fig. 5a and b; Supplemental Fig. S2). Although the tilt-pair analysis and close correlation with models of the holoenzyme soluble region give confidence in the overall validity of the reconstructions, the part of the model corresponding specifically to this asymmetric density must be considered to be of lower reliability. Heterogeneity in the data, specifically for views of this region, is the most likely reason for failure to unambiguously resolve it. One possible source of heterogeneity is mobility of individual subunits, with different room-temperature conformations becoming trapped by rapid freezing. Although this low reliability necessarily means assignment of subunits to this density must be speculative, subunits H and C are the only plausible candidates. Based on biochemical [26], [27], [49] and chemical cross-linking [28] data, this weak density should be subunit H. From the analysis of V_1_ and V_1_^+ C^ composition (Fig. 2), it is clear that subunit H is present at stoichiometric levels equivalent to those in the holoenzyme. Although the longest dimension (100 Å) of the subunit H crystal structure [11] matches that of the density at the base of V_1_^+ C^, overall, it is a poor fit, extending beyond the bounds of the reconstruction contoured at the level shown (Supplemental Fig. S4a′ and b′). Consequently, we can speculate that subunit H can link S1 to the axle, but some uncertainty must remain because of the poor quality of the data specifically relating to this region.

The apparent absence from the V_1_ reconstruction of a subunit H density cannot be explained by its loss from the preparation, since SDS-PAGE shows levels to be identical in specimens of V_1_ and V_1_^+ C^ used for EM and in the V-ATPase (Fig. 2). A 3D reconstruction of *Manduca* V_1_ from negative-stain data (Supplemental Fig. S1d) does show a density in a similar position at its base to that of the putative H subunit in the V_1_^+ C^ cryo-EM reconstruction. This negative-stain model is essentially indistinguishable from a previously published *Saccharomyces* V_1_ model where the density in question was assigned to H [33]. It accounts for ~ 10% of the total volume of the ~ 660-kDa complex and must accommodate the 55-kDa H subunit. Visible in individual classes (Supplemental Fig. S1b), it is presumably rendered immobile by the staining process or by adhesion to the carbon support. There are several reasons why information relating to the putative H subunit density in the cryo-EM reconstruction could be missing or lost. Misalignment of particles (perhaps because of the pseudo-3-fold symmetry of V_1_) could lead to decreased signal. However, this seems unlikely, given that the methods used here have resolved relatively minor asymmetrical features of the stator filaments and catalytic hexamer. Degradation or damage to the H subunit could introduce heterogeneity that would lead to poor resolution but is not evident from the protein analysis shown in Fig. 2 that shows intact, stoichiometric H. Hildenbrand *et al.* have suggested that failure to resolve stator components E, G and C in a cryo-EM model of yeast V_1_ may be due to their detachment during cryo-grid freezing [50]. Similar detachment of H seems unlikely, given the strength of its interaction with V_1_ during the purification process and the speed of transition from liquid to ice during cryo-freezing (< 1 ms). In this study, there is no evidence for EG dissociation. Consequently, heterogeneity resulting from conformational variability remains as a plausible explanation.

If we are confident that both the V_1_ and V_1_^+ C^ specimens do contain similar high levels of the H subunit (Fig. 2d–f), then the question also remains about the location of H in the V_1_ cryo-EM model. If contouring of the V_1_ model is brought closer to the level of noise, a density is evident between the ends of S1 and S2 that can accommodate the crystal structure of H [11] (Supplemental Fig. S4g′ and h′). Similarly, at this contouring level, a density with the same shape and dimensions as subunit C [12] is visible in the consensus position linking S2 and S3 in the V_1_^+ C^ model (Supplemental Fig. S4c, d, c′ and d′). As with the putative subunit H density in V_1_^+ C^, the weakness of these densities is also consistent with either conformational variability or low stoichiometry in the particles on the grid. Given the observed high level of H in V_1_ and C in V_1_^+ C^, the former explanation is more likely. If correct, comparison of V_1_ and V_1_^+ C^ suggests that H can adopt either of two minimal energy configurations: aligned between S1 and S2 in a position similar to that in the holoenzyme or rotated about S1 to contact the rotor axle. It is not clear why re-association with C-His_6_ would impose this second conformation, and no biological significance can be attached to the difference in the visibility of H in the two reconstructions. Its resolution in V_1_^+ C^ (albeit poor) is almost certainly a result of the better overall resolution of this model and not a structural consequence of C-His_6_ binding.

## Discussion

The detailed models of V_1_ after *in vivo* inactivation described here give new insight into structural changes that accompany V-ATPase regulation. Previous V_1_ reconstructions had much less detail, probably because of the limitations of the negative-staining method [32], [33] or decomposition due perhaps to freeze–thawing of the sample or during grid preparation [50]. In this study, it was also found that conventional refinement schemes were incapable of escaping from the apparent local optimum of the negative-stain model, perhaps hampered by the small size and pseudo-3-fold symmetry of the complex at low resolution. However, our use of maximum-likelihood classification tools [39] gave significantly improved 3D models, probably as a consequence of increasing the homogeneity of the final datasets used to generate the reconstructions by eliminating damaged, degraded or disassembled particles. Unsupervised multiple refinements from an essentially spherical starting model [the V_1_ reconstruction from negative-stain data (Supplemental Fig. S1d) filtered to 100 Å] yielded a consistent reconstruction in excellent agreement with the V_1_ region of the intact V-ATPase. The *Manduca* V-ATPase reconstruction was not used at any stage during data processing in order to ensure that no model bias was introduced. This allows a bias-free comparison between the V_1_ domains and the V-ATPase complex to be made. Tilt-pair analysis confirmed the validity and absolute hand of the V_1_^+ C^ model, therefore also indirectly validating the V-ATPase model.

### Mechanical stiffness of the V-ATPase stator filaments

Full-length stator filaments projecting from V_1_^+ C^/V_1_ that are largely superimposable with those of the holoenzyme were surprising, given that the ends of S2 and S3 are completely unsupported. Because cryo-EM freezing occurs rapidly (~ 10^5^ °C/s [51]), reconstructions are thought to show the room-temperature conformation. Conformational variations trapped by freezing will show as heterogeneity in the data, resulting in the variable regions to become averaged out and consequently absent from the final reconstruction. The appearance of V_1_ stator filaments with essentially the same positions, length and curvature as the corresponding structures in the holoenzyme indicates firstly that, at the point of freezing, each was in a single minimal energy configuration. Secondly, it indicates that the S1–S3 filaments in the holoenzyme are subject to very little, if any, flexing or bending as a consequence of attachment to the H-*a*_N_-C collar. Only S2 undergoes a 7° change in angle as a consequence of release from its “high-avidity” [52] junction with *a*_N_ and C (Supplemental Fig. S3). Thus, it appears that the EG stator filaments have inherent stiffness, a property also proposed for the single stator filament of the F-ATPase [17]. A capacity for some radial flexing by the A-ATPase EG filament has been suggested on the basis of normal mode analysis and comparison of prokaryotic EG crystal structures [34]. This could accommodate sequential conformational “breathing” of each AB unit during the catalytic cycle. Our observations are consistent with the concept of the stator filaments acting as flexible rods, but beyond the small change in angle of S2, those do not report on the potential for azimuthal flexing presumed to be necessary for a role in elastic power transmission. It is also interesting to note that stator filament S1 retains the same more vertical configuration in deactivated V_1_ as in the holoenzyme despite the absence of *a*_N_-H-C collar to which it attaches. The cause-and-effect relationship is uncertain, but adoption of this shape correlates with subunit H binding and with proximity to the AB unit in the “open” state.

### V-ATPase regulation and silencing ATP turnover by V_1_

Our 3D reconstructions suggest refinement of a model for the rearrangements leading to domain dissociation. Although the signals that trigger V_1_ detachment and ATPase deactivation remain uncertain, there is evidence that phosphorylation of subunit C is a major factor in some organisms [23], [24]. Alternatively, an unstable but normally transient ADP-bound conformation could persist when ATP/ADP ratios are low [29]. From the V_1_ models here, there is now at least much more detailed information about the rearrangements occurring as a consequence of these signals. Firstly, each of the three stator filaments occupies the same position in the V_1_ and V_1_^+ C^ reconstructions, indicating that subunit C binding has no direct influence on their conformation. Secondly, S1 and S3 are identical in V_1_/V_1_^+ C^ and the holoenzyme, indicating that integration into the collar of the intact enzyme does not influence their conformation. Changes to the positions of *a*_N_, C and H that occur with domain separation must therefore take place around an essentially invariant stator filament framework. Only the position of S2 shows a modest difference between V_1_/V_1_^+ C^ and the holoenzyme, suggesting a change in its angle as a consequence of detachment. It is likely that the trigger for domain dissociation is not subunit C release per se but, rather, destabilisation of the low-affinity but high-avidity junction between *a*_N_, S2 and the “foot” of subunit C [52]. Although there is currently direct evidence of controlled domain dissociation only in the yeast and insect systems, regulation of V-ATPase activity via pH sensing [53] or Ca^2 +^-calmodulin binding [54] by subunit *a* could also occur. There is therefore the potential for regulatory signals to be channelled through either wing of the H-*a*_N_-C collar to the crucial junction with S2.

**Fig. 7:**
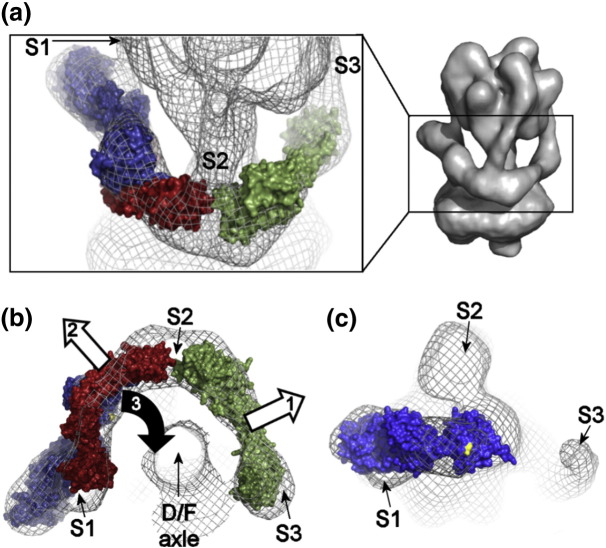
Model for V-ATPase domain dissociation. (a) The H-*a*_N_-C collar in the V-ATPase holoenzyme plays a central role in dissociation *in vivo*. The electron density map of the holoenzyme [7] is shown as mesh fitted with crystal structures of H (blue), bacterial *a*_N_ (red) and C (green). Positions of the stator filaments S1–S3 are indicated. The corresponding region in the holoenzyme model is boxed, right. (b and c) Rearrangements in the collar during detachment of V_1_. In (b), the collar of the intact holoenzyme is viewed from the membrane. (1) Signals acting on subunit C cause the low-affinity “foot” end to detach from the high-avidity junction with S2 and *a*_N_. Ultimately, subunit C may detach completely from the collar. (2) Interaction between S2 and *a*_N_ is destabilised, and *a*_N_ binding to H is weakened. V_1_ becomes detached from V_o_. (3) Domain separation gives H the mobility about S1 needed to make contact with the D/F axle. This allows it to adopt (perhaps transiently) the position found in the V_1_^+ C^ reconstruction shown in (c) [as mesh at 1.5σ level, from the same perspective as in (b)]. The residue coloured yellow in the H crystal structure has been shown to cross-link to F only in detached V_1_[28].

An outline model of the dissociation process is shown in Fig. 7. Subunits C, H and *a*_N_ are maintained in “active” conformations in the collar of the intact V-ATPase (Fig. 7a and b) by extensive H-*a*_N_, H-EG, *a*_N_-EG and C-EG contacts (Ref. [3] and references therein for detailed discussion). S1 and S2 are highly likely to be physically linked by *a*_N_ because this “half-collar” feature is also present in the A-ATPase that lacks C, H and S3 [3]. We propose that the “low-affinity” subunit C–S2 interaction is required to maintain S2 contact with *a*_N_. Shape changes in C result in its detachment from S2, in turn, causing release of *a*_N_ (Fig. 7b). Without its S2 contact point, *a*_N_ is able to recoil from H, resulting in the detachment of V_1_. Unconstrained by *a*_N_, a mobile H could make transient contact with the rotor axle as suggested by the V_1_^+ C^ reconstruction (Fig. 7c). Contact between the end of the axle and the putative H density is consistent with ATPase inhibition by recombinant H [27] and cross-linking between H and axle subunit F in deactivated *Saccharomyces* V_1_ [28]. However, the benzophenone cross-linker used in the latter study can be photoactivated repeatedly until it forms a stable cross-link; hence , it is particularly efficient at trapping the type of transient interaction suggested by the data here. From its position in the V-ATPase, the putative H density is tilted ~ 40° and rotated ~ 50° towards the mid-line of the complex. The latter movement is feasible based on the position of the proposed H density in reconstructions from negative-stain data [33] (Supplemental Fig. S1). Subunit H is an α-solenoid-type protein with extensive armadillo/HEAT repeats in its N-terminal domain [11] known to confer flexibility [55], [56]. The 7° shift in angle of S2 after dissociation could indicate that it is bent or flexed in the holoenzyme but, equally, could be a rigid-body movement reporting on a difference in AB conformation in detached V_1_ compared to the intact enzyme.

How might these rearrangements prevent ATP hydrolysis? Based on the arrangement of subunit A and B densities in the V_1_ map (Fig. 6), the catalytic unit adjacent to S1 is trapped in the “open” state described by the binding change mechanism. The other two active sites are likely to be locked in ADP-bound states, given that ~ 2 ADP are present per V_1_ [29]. Even in the presence of ATP, transition of the “open” site to the “tight” state is blocked either because contact between H and the axle prevents stepping of the V_1_ motor or because binding of H to S1 (or to the axle) imposes a conformation on S1 that constrains movement of the AB subunits and therefore obstructs catalysis. Pertaining to this point, *Saccharomyces* V_1_ assembled in the absence of H retains strong (if short-lived) Mg·ATPase activity that is inhibited by ~ 76% by restoring expression of only the N-terminal domain of H [27]. This region, which interacts with S1, would not be long enough to link S1 to the rotor axle. Hence, binding to S1 alone may be sufficient to impose a strong inhibitory effect on V_1._ Other mechanisms may also play a role: the rapid diminishing of the V_1_ Mg·ATPase activity even in the absence of H could stem from ADP inhibition. Alternatively, rearrangement of the F subunit could switch off ATPase activity via an unresolved mechanism analogous to that proposed for the ε-subunit in the bacterial F_1_F_o_-ATPase axle [30], [57]. However, improved resolution would be needed to allow reliable assignment of a position for F in the V_1_ model.

Binding of subunit C to deactivated V_1_ must precede V_1_–V_o_ functional re-association, and we can speculate about the steps in domain re-engagement that this initiates. The fit of C to reconstructions of *Manduca* and *Saccharomyces* V-ATPases is optimal with its high-affinity “head” in contact with S3 [7], [58]. The initial binding of C to V_1_ is therefore most likely to be to S3, leaving the N-terminal His_6_-tag available for binding to an affinity resin (as shown in Fig. 2a). After binding, C would retain the conformational flexibility evident from small-angle X-ray scattering studies on the EGC complex [20]. This could explain its apparently disordered state in our V_1_^+ C^ reconstruction. Immobilisation by binding of the low-affinity “foot” to S2 would not be possible because the change in S2 angle after domain separation introduces ~ 20 Å greater spacing between the filament ends that cannot be bridged by C. Interactions between V_1_ and V_o_ via S1/H binding to *a*_N_ would lead to re-association only if C was available to stabilise the *a*_N_-S2-C junction. Hence, binding C in an “active” conformation would be a key step in priming V_1_ for functional re-coupling. S2 must move back to the position seen in the holoenzyme in order to allow C to contact both S3 and the *a*_N_-S2 junction. This S2 repositioning could be driven by conformational changes in the AB complex associated with catalysis.

The V-ATPase is a dynamic and complex molecular machine. Key to understanding its mechanisms of operation and regulation are the ability to map the sequence of conformational changes involved in these processes. The results presented here are steps towards this goal. It is clear that domain dissociation must result from a sequence of subtle effects on the protein–protein interactions that maintain mechanical coupling. In addition, models of inactivation in which the H subunit locks the rotor of the V_1_ motor appear to be over-simple: H may have more conformational variability than previously thought. The way in which ATP turnover is inhibited within the dissociated V_1_ therefore needs further study to establish the exact roles played by H and perhaps F. The apparent stiffness of the stator filaments must also be taken into account when trying to understand the basis for elastic coupling between asymmetrical ATPase and H^+^ pump functions.

## Materials and Methods

### Electron microscopy

*M*. *sexta* V_1_ was extracted and purified as previously described [35]. Negative-stain EM was used to check purity and monodispersity and to collect data for an initial reconstruction (Supplemental Experimental Procedures; Supplemental Fig. S1). Cryo-EM grids were prepared by applying 1 mg/ml V_1_ to Quantifoil and Lacey grids that had been glow-discharged for 30 s before being squeeze-blotted from both sides for 7 s and plunge-frozen in liquid ethane. For grids of V_1_^+ C^, V_1_ was mixed with recombinant C for 1 h at 4 °C (1:5 V_1_:C molar ratio) and then placed on Lacey grids that had been glow-discharged before being blotted for 7 s and plunge-frozen in an FEI Vitrobot. Grids were imaged at 69,000 nominal magnification in a Tecnai F20 microscope operating at 200 kV. Micrographs were recorded on an Ultrascan 4000 × 4000 CCD camera, using a range of defocus values (1.5–3.5 μm, determined using CTFFIND3). Micrographs showing signs of astigmatism or drift were removed. In total, 42,000 particles were selected using BOXER [59] and extracted as 128 × 128 pixel images with a final pixel size corresponding to 2.18 Å, which were subsequently reduced to 64 × 64 pixels with pixel size of 4.4 Å. Maximum-likelihood refinement removed poor-quality particles, leaving 16,500 V_1_ and 10,746 V_1_^+ C^ particles contributing to the final refined models.

### Image processing

Particle normalisation, projection matching refinement of the negative-stain model and MLF3D classification were performed using standardised procedures from the XMIPP package [40]. Particles were normalised to have zero mean density with unity standard deviation in the area outside a centred circle with a radius of 30 pixels. A negative-stain model (Supplemental Fig. S1d) was low-pass filtered to 100 Å and used to generate initial reference seeds for MLF3D classification. Different seeds were obtained by dividing the particle set into four random subsets and performing a single iteration of MLF3D refinement of the filtered model against each of the subsets. Subsequent MLF3D multi-reference refinements of the four seeds against all particles were stopped after 25 iterations, and particle assignments to one of the four classes were based on the maximum of their probability distributions. All MLF3D refinements were performed without CTF correction and with an angular sampling rate of 10°. Subsequent refinement of the predominant class was performed using the 3D auto-refine procedure in RELION [41]. Resolution estimates were based on gold-standard Fourier shell correlations, using the 0.143 criterion [42]. Absolute hand and validation of the model was performed using the tilt-pair analysis method [43]. Crystal structure fitting and density map superpositioning was performed using Chimera [60]. Display figures were generated using PyMOL [61]. The rotational symmetry within the V_1_ domain was calculated using the Test-Rotation-Symmetry command in Imagic-5 [62]. V_1_ and V_1_^+ C^ maps have been deposited in the EMDataBank with accession codes 2318 (V_1_) and 2317 (V_1_^+ C^).

### Biochemical analysis of V_1_ and V_1_^+ C^

Binding of subunit C to V_1_ was confirmed by co-precipitation of V_1_ with His_6_-tagged recombinant subunit C after capture on IMAC resin. Purified V_1_ [~ 20 μg in 20 mM Tris–HCl and 100 mM NaCl (pH 7.9)] was incubated with a 5-fold molar excess of recombinant subunit C-His_6_ at 4 °C for 3 h before binding to “Talon” Co^2 +^-bound resin (BD Biosciences) for a further 1 h. Alternatively, the C-His_6_ was pre-bound to the Talon resin before addition of V_1_, but this did not affect binding. As controls for non-specific binding, V_1_/C-His_6_ binding in the presence of 300 mM imidazole and V_1_ binding in the absence of C-His_6_ were also tested. After binding, the Talon resin was washed 3 × with the abovementioned buffer before re-suspension in SDS-PAGE sample buffer and analysis on 4–12% polyacrylamide gradient gels (Invitrogen). Gels were routinely stained with colloidal Coomassie stain (Severn Biotech Ltd.) that does not detect the D subunit, which could instead be visualised by silver staining (Pierce Silver Staining Kit; Thermo Scientific).

Efficiency and stability of C-His_6_ binding was estimated by re-purifying the V_1_^+ C^ complex by size-exclusion chromatography and comparing the relative levels of retained C-His_6_ to those in the V-ATPase holoenzyme. The reconstituted sample was passed through a Superose-6 column (23 ml bed volume; GE Healthcare) linked to an Akta Purifier, developed with 20 mM Tris–HCl, 100 mM NaCl, 10 mM KCl and 2 mM 2-mercaptoethanol at pH 8.0 at a flow rate of 0.25 ml/min. Fractions (0.5 ml) were analysed by SDS-PAGE as mentioned above. The column was calibrated by monitoring the elution of standards [thyroglobulin (660 kDa), ferritin (445 kDa), catalase (250 kDa), aldolase (148 kDa) and albumin (66 kDa)].

To assess the relative levels of C and H subunits in V_1_ and V_1_^+ C^ preparations, we quantified the corresponding bands on stained SDS-PAGE gels digitised using Syngene G-Box or Fujifilm LAS-3000 scanners using Aida v4.15 image analysis software. C and H levels were expressed relative to the intensity of the subunit A band. Gel lanes needed to be loaded with < 10 μg protein and electrophoresis run until the 25 kDa marker (BioRad “Precision Plus” marker set) reached the bottom of the gel in order to achieve the spatial separation of B and H subunits required for reliable quantitation of H.

The following are the supplementary data related to this article.

Supplementary video

Supplementary material
