# Supplementary material for: Subunit Positioning and Stator Filament Stiffness in Regulation and Power Transmission in the V1 Motor of the Manduca sexta V-ATPase
Source: J Mol Biol. 2014 Jan 23;426(2):286–300. doi: 10.1016/j.jmb.2013.09.018 (PMC3899036; doi:10.1016/j.jmb.2013.09.018)
Supplement: Supplementary file 2 — Supplementary material [file mmc2.doc]

**Subunit Positioning and Stator Filament Stiffness in Regulation and Power Transmission in the V_1_ Motor of the *Manduca sexta* V-ATPase**

**Stephen P. Muench *et al***

**Supplemental Methods: Negative stain electron microscopy**

Grids were prepared by applying 3ul of protein solution (~40µg/ml) onto a carbon-coated copper grid that had previously been glow discharged for 40 minutes under a UV lamp. The grid was then stained with 1% uranyl acetate. Grids were imaged at 40k magnification using a Jeol 1200EX microscope fitted with a LaB6 filament operating at 80 kV. Micrographs were recorded on Kodak SO163 film, which was developed in D19 developer and scanned using a Nikon Coolscan 9000 scanner at 1.56Å/pixel.

In total ~54204 particles were selected using BOXER. These images were binned by a factor of 2 (resulting in an Å/pixel value of 3.12), normalized and band pass filtered to remove low and high spatial frequencies (low = 0.03 & high = 0.65). The particles were centred, masked and classified in the IMAGIC-5 suite of programs resulting in a ‘crude’ set of reference images, which were used for multi-reference alignment. The classes produced by the aligned data were significantly improved and representative classes were used for a further stage of multi-reference-alignment. This process was iterated until no further improvement in the resulting classes could be observed. Those particles which aligned poorly, were unstable during classification or produced clearly degraded or non-representative views of the V_1_ complex were removed resulting in a data set of the ~20000 best particles. Two distinctive classes were produced which could be identified as the top view, which displayed clear 3 fold symmetry, and side views which displayed clear 2-fold symmetry in the catalytic domain (Suppl. Fig S1*B*, *C*).

A 3-D reconstruction was generated in EMAN using the previously solved intact *M. sexta* V-ATPase reconstruction which was cropped so only the V_1_ catalytic domain remained. This starting model was then low pass filtered to 60Å to try to remove unwanted model bias. The resulting 3D reconstruction showed clear differences to the starting model. A significant difference was the lack of any stator connections protruding from the base of V_1_. This is likely to be due to staining artefacts such as flattening. In addition extra density is found at the base of V_1_ which is not present in the holo-enzyme and must represent the H subunit since all other subunits are accounted for and subunit C was not added to this reconstruction.

Crystal structure fitting to 3-D reconstructions was performed using Chimera. Figures were produced using PyMol.

**Supplemental Figures**

**Figure S1. Electron microscopy of negatively-stained V_1_**

*A*, typical region of negatively stained V_1_ domains showing a characteristic roughly spherical shape with low background noise. Grids were stained with uranyl acetate. Class averages of the V_1_ domain in side *(B)* and top views *(C)*. The scale bar represents 50 Å. *D,* negative stain reconstruction of the V_1_ domain as seen from the side, rotated 120° to the right, and the top and bottom.

**Figure S2. Comparison of V_1_^+C^ and V_1_ reconstructions.**

*A*-*C*, side views of the V_1_^+C^, V_1_ and holoenzyme reconstructions, respectively. *D*, *E, F*, corresponding views after rotation of each model by 180^o^. *G*-*H*, bottom views of V_1_^+C^ and V_1_ respectively. Stator filaments S1-S3 are labelled as in reference 11 and the asterisk in *A*-*C* shows the position of the rotor axle. Maps are contoured at 1.5σ. The key difference between V_1_^+C^ and V_1_ is the absence of the subunit H density in the V_1_ reconstruction apparent at this contouring level (see main text for discussion).

**Figure S3. Subunit E/G fitting and stator filament angles in V_1_^+C^ and holoenzyme.**

*A*. Crystal structures of different forms of the EG heterodimer were fitted to S1-S3 using Chimera. Green: pdb 3V6I (*Thermus thermophilus* ‘PS2’ form)^30^; Blue: pdb 3K5B^43^; Magenta: EG part of pdb 4DL0^44^; Cyan: EG part of pdb 4EFA^44^. *B*, details of stator filaments S1-S3 in the *Manduca* V-ATPase and *C*, in the V_1_^+C^ reconstruction. In *D*, EG heterodimers fitted to equivalent stator filaments in each reconstruction are superimposed. Only S2 shows a significant shift in position between V_1_ and the holoenzyme.

**Figure S4. Subunit C and H densities in V_1_^+C^/V_1_ at lower sigma values.**

*A*-*B*, the V_1_^+C^ reconstruction contoured at 1.5σ, with the same views also at 0.75σ (*C*-*D*). Crystal structures of subunits H (blue: pdb 1HO8^47^) and C (green: 1U7L^16^) are shown fitted to the corresponding semi-transparent models (*A’*-*D’*). The density accommodating subunit C that links S2 to S3 is only evident at the lower contouring level shown in *C* and *D*. *E*-*F*, *G*-*H,* the V_1_ reconstruction at 1.5σ and 0.75σ, respectively. In *G’* and *H’*, subunit H (blue) has been fitted to the density linking S1 and S2 evident in V_1_ only at the lower contouring level.


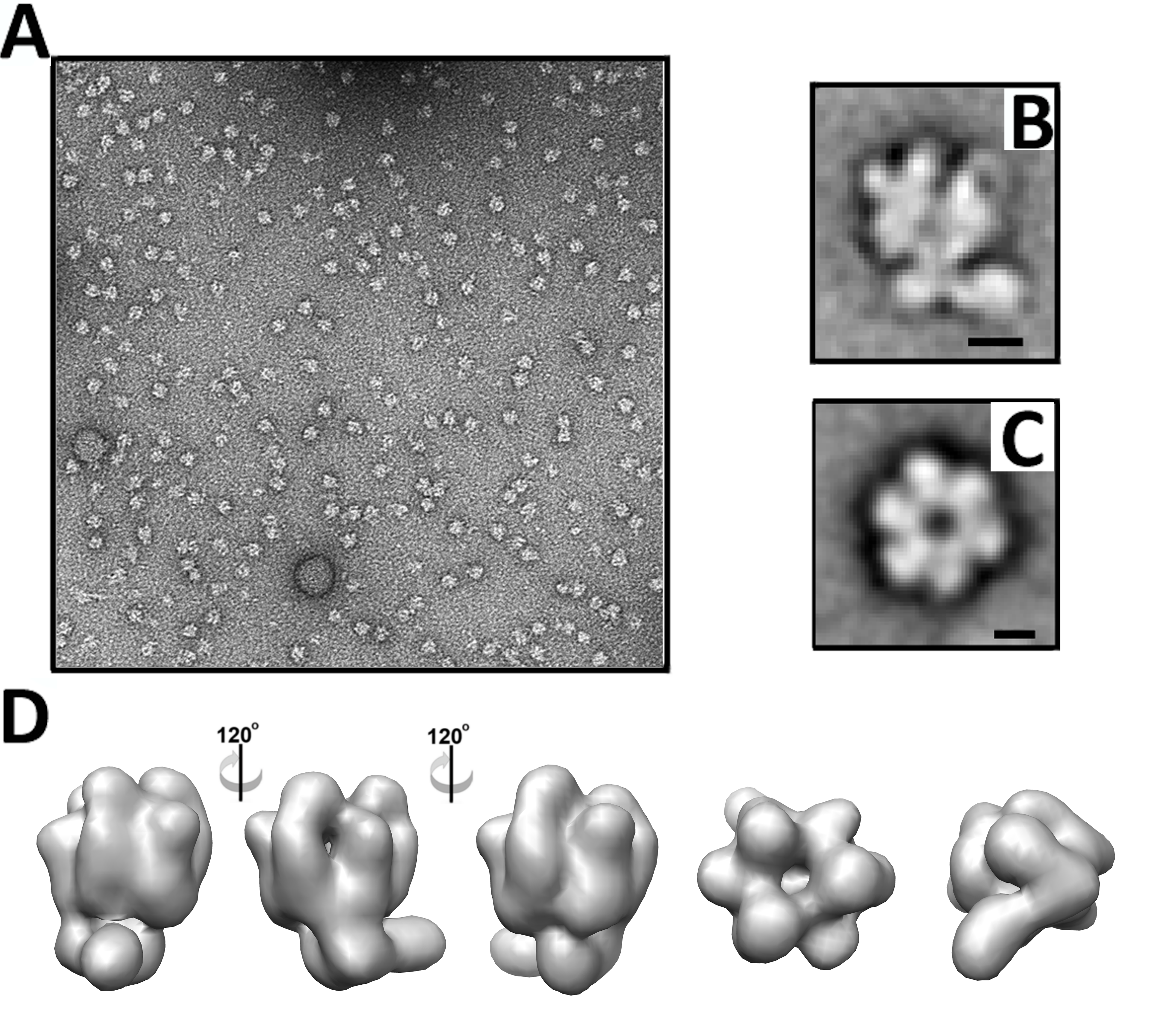


**Supplemental Figure S1**

**
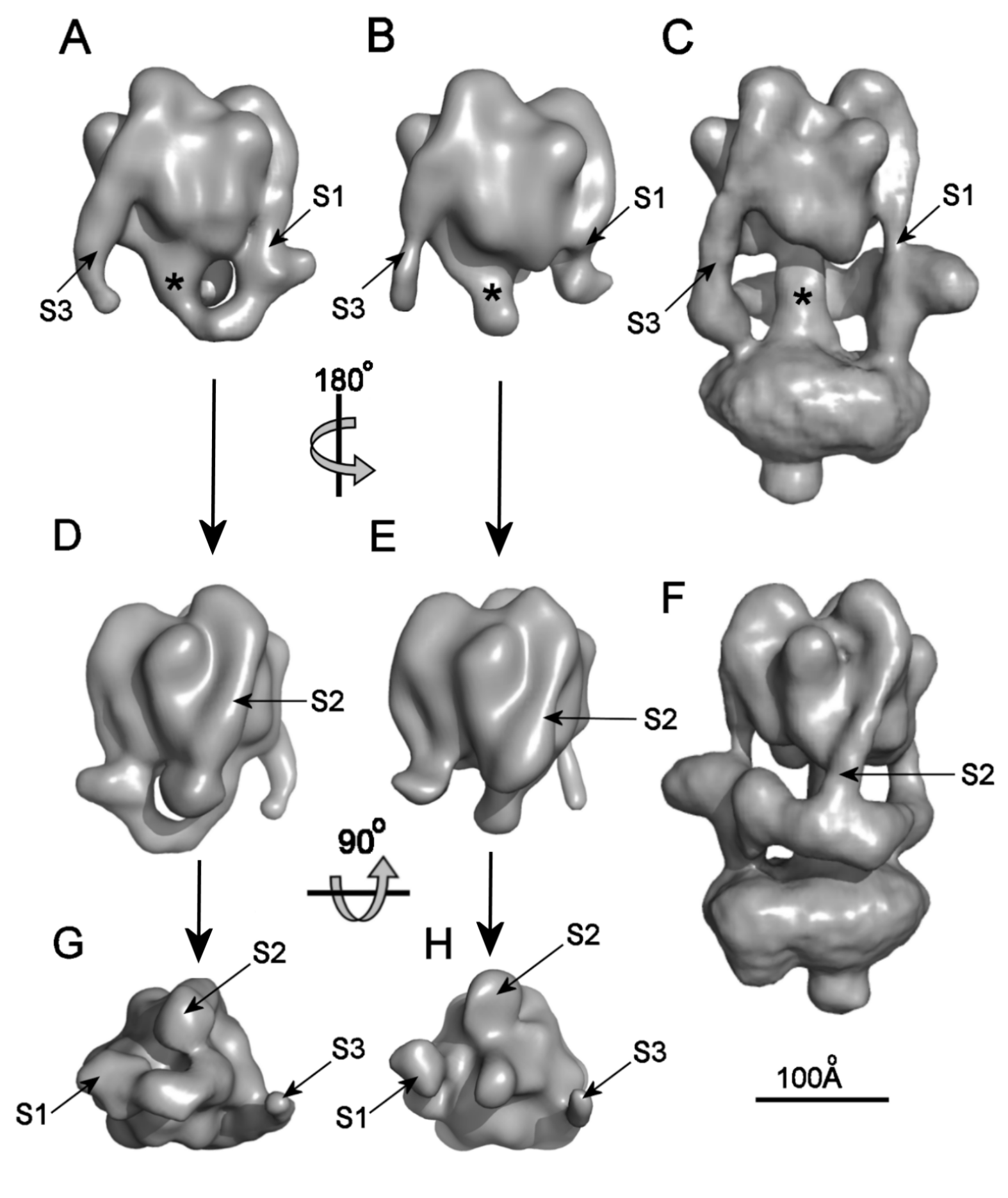
**

**Supplemental Figure S2**

**Supplemental Figure S3**

**
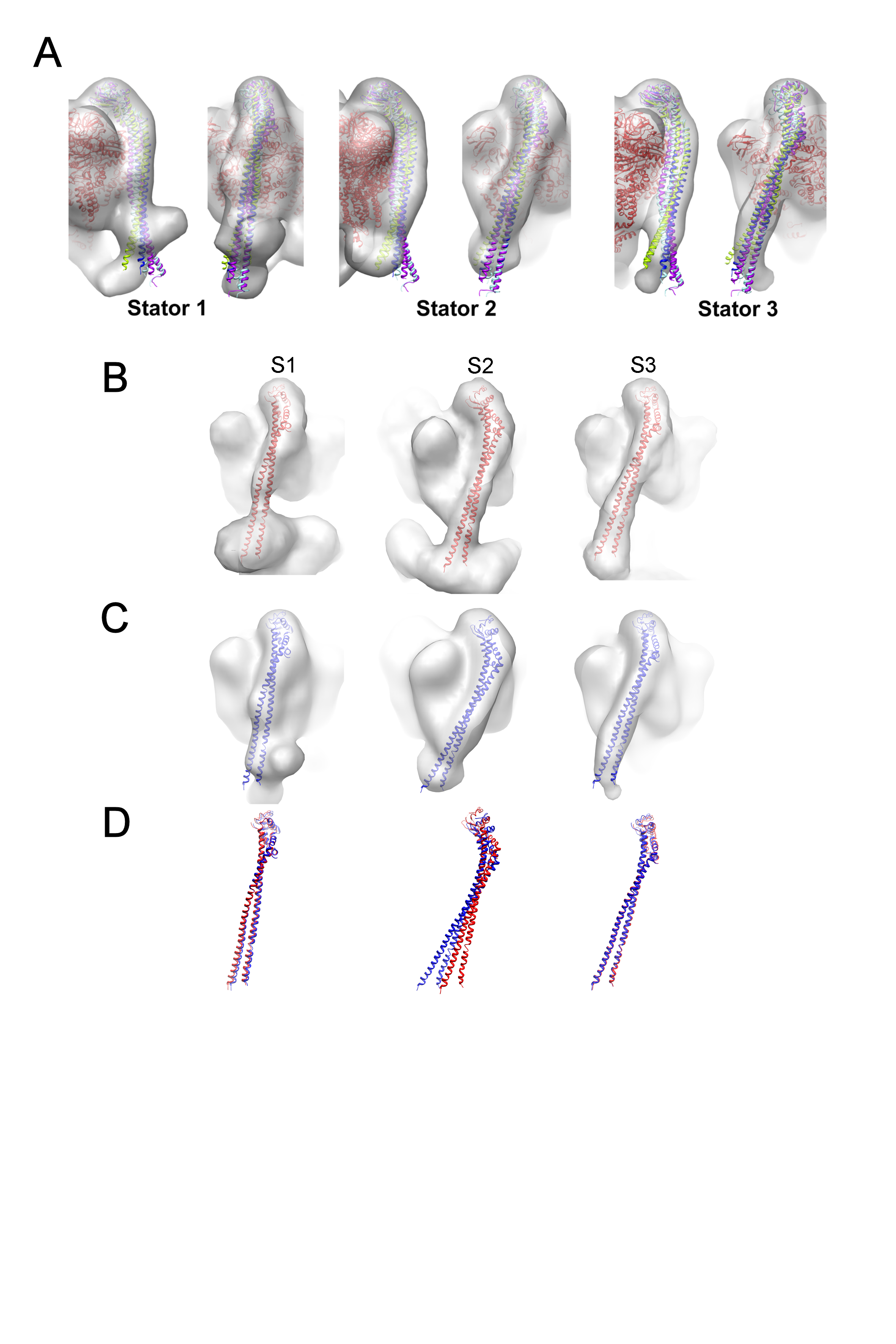
**

**
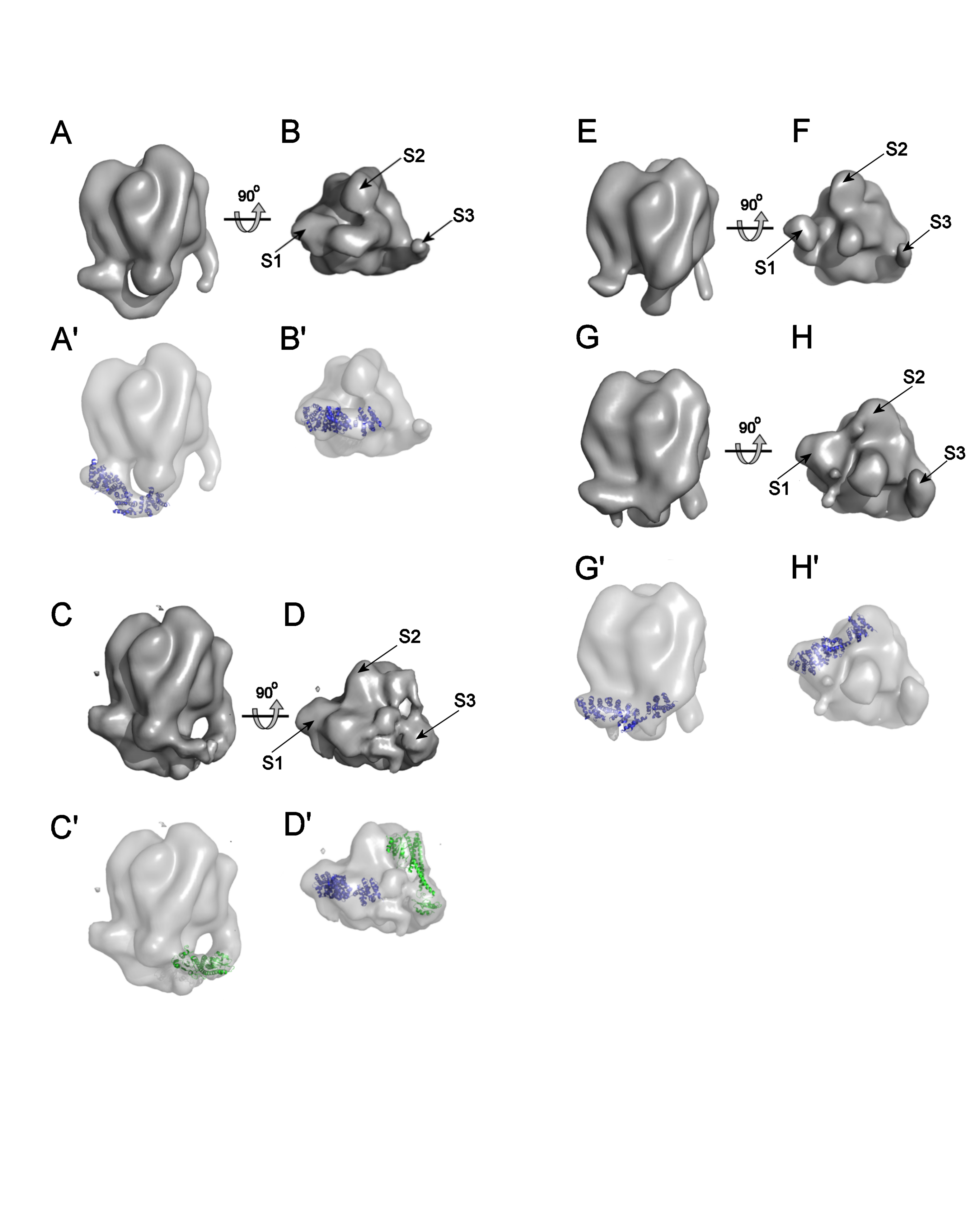
**

**Supplemental Figure S4**
